# Seed priming with potassium nitrate alleviates the high temperature stress by modulating growth and antioxidant potential in carrot seeds and seedlings

**DOI:** 10.1186/s12870-024-05292-1

**Published:** 2024-06-26

**Authors:** Muhammad Mahmood ur Rehman, Jizhan Liu, Aneela Nijabat, Ibtisam M. Alsudays, Muneera A. Saleh, Khalid H. Alamer, Houneida Attia, Khurram Ziaf, Qamar uz Zaman, Muhammad Amjad

**Affiliations:** 1 School of Agricultural Engineering, Jiangsu University, Zhenjiang, 212013 China; 2 Institute of Horticultural Sciences, University of Agriculture Faisalabad, Faisalabad, 38040 Pakistan; 3 Department of Botany, University of Mianwali, Mianwali, 42200 Pakistan; 4 Department of Biology, College of Science, Qassim University, Burydah, 52571 Saudi Arabia; 5 Department of Biology, College of Sciences, Taif University, P.O. Box 11099, Taif, 21944 Saudi Arabia; 6 Biological Sciences Department, Faculty of Science and Arts, King Abdulaziz University, Rabigh, 21911 Saudi Arabia; 7 Department of Environmental Sciences, The University of Lahore, Lahore, 54590 Pakistan

**Keywords:** Carrot growth, Heat stress, Potassium nitrate, Reactive oxygen species, Seed treatments

## Abstract

Early season carrot (*Daucus carota*) production is being practiced in Punjab, Pakistan to meet the market demand but high temperature hampers the seed germination and seedling establishment which cause marked yield reduction. Seed priming with potassium nitrate breaks the seed dormancy and improves the seed germination and seedling growth potential but effects vary among the species and ecological conditions. The mechanism of KNO_3_ priming in high temperature stress tolerance is poorly understood yet. Thus, present study aimed to evaluate high temperature stress tolerance potential of carrot seeds primed with potassium nitrate and impacts on growth, physiological, and antioxidant defense systems. Carrot seeds of a local cultivar (T-29) were primed with various concentration of KNO_3_ (T_0_: unprimed (negative control), T_1_: hydroprimed (positive control), T_2_: 50 mM, T_3_:100mM, T_4_: 150 mM, T_5_: 200 mM, T_6_: 250 mM and T_7_: 300 mM) for 12 h each in darkness at 20 ± 2℃. Seed priming with 50 mM of KNO_3_ significantly enhanced the seed germination (36%), seedling growth (28%) with maximum seedling vigor (55%) and also exhibited 16.75% more carrot root biomass under high temperature stress as compared to respective control. Moreover, enzymatic activities including peroxidase, catalase, superoxidase dismutase, total phenolic contents, total antioxidants contents and physiological responses of plants were also improved in response to seed priming under high temperature stress. By increasing the level of KNO_3_, seed germination, growth and root biomass were reduced. These findings suggest that seed priming with 50 mM of KNO_3_ can be an effective strategy to improve germination, growth and yield of carrot cultivar (T-29) under high temperature stress in early cropping. This study also proposes that KNO_3_ may induces the stress memory by heritable modulations in chromosomal structure and methylation and acetylation of histones that may upregulate the hormonal and antioxidant activities to enhance the stress tolerance in plants.

**Supplementary Information:**

The online version contains supplementary material available at 10.1186/s12870-024-05292-1.

## Introduction

Food security is a most challenging concern of the current era and food production must increase up to 56% by 2050 to meet the basic dietary need of growing population [1]. Unfortunately, global climate change with extreme weather events threatens the agricultural production [2]. Additionally, off season farming of food crops is being practiced in various countries to increase the profit and meet the market demand but in arid and semi-arid regions, production of off season farming is considerably low rather regular cropping due to the raising temperature [3]. Previous studies reported more than 50% yield reduction of vegetables exposed to frequent heat waves in both regular and off season farming [4, 5]. Cool season vegetables are highly sensitive to high temperature and high temperature hampers the growth from seed germination till maturity [6, 7]. Seed germination is very crucial among all growth stages because low germination rate lowers the population density per unit area that ultimately reduced the yield [8]. Seed germination is a complex process involves three main phases such as water imbibition, activation of physiological processes and gene expression, and radicle emergence [9]. Water uptake by cytoplasmic compounds and macromolecules hydrolysis occur in initial 24 h that triggers the ATP and protein synthesis followed by radicle emergence after 48 to 72 h of imbibition [10]. Thus, all the physiological and molecular events in the germinating seed not only depend on water imbibition, also linked with several physical factors including temperature [11]. Optimum temperature range varies among species but temperature beyond the threshold value causes damage to embryo or complete cell death in various crops [12]. Low temperature causes chilling stress and is more critical during seed germination which halts the water imbibition and inhibits the seed germination and growth from early seedling establishment till maturity [13]. Level of chilling stress tolerance as per the exposure time and growth stage varies among different plant species but the mechanism to avoid or tolerate the stress is almost similar [14–17]. Similarly, high temperature has become a major constraint that hinders the plant growth from seed germination to onwards growing stages till harvest. High temperature stress in plants is also linked with drought stress due to elevated transpiration level to avoid the high temperature by cooling effect [18]. Previous studies reported that plant responses towards heat stress are regulated by several genes and signaling cascades. Heat shock transcription factors are conserved in most of the plant species and their regulation for heat responsive genes play crucial role in high temperature stress tolerance [19].

Carrot is a temperature sensitive winter crop and temperature > 35℃ caused 47% yield losses by inhibiting seed germination and seedling growth at subsequent growth stages in regular cropping [4, 6]. Initially high temperature faster the water imbibition rate and increase the seed moisture and osmotic level which adversely effects embryonic potential [20]. Additionally, high temperature causes irreversible damages to cellular membranes by altering the quantity and quality of several structural compounds [21]. This allows an uncontrolled ion outflow, and leakage of cellular contents and it also induces the overproduction of reactive oxygen species (ROS) which lower the seed germination potential [22, 23]. Moreover, nutrient availability facilitates several electrochemical and catalytic functions but high temperature slow down the nutrient uptake and their utilization [24]. Thus, high temperature intensification requires optimum nutrient availability and use efficiency to avoid high temperature stress [25]. A recent study suggested that exogenous application of essential micromolecules through priming, foliar sprays, and soil fertilization enhanced the abiotic stress tolerance in plants [26].

Seed priming with essential inorganic salts not only facilitates uniform and rapid seed germination and radicle emergence, also promote seedling growth at subsequent stages by regulating the several healthy physiological activities in the seed before germination [27–32]. Generally, seed priming facilitate the physiological stages (imbibition, activation, and rehydration) of germination. Initially, seed has low water content and it imbibes water rapidly which leads to activation of several metabolic activities at cellular and sub-cellular level. Synthesis and activation of several proteins and antioxidants, DNA repairing, formation of new mitochondria, and hydrolysis of reserve food contents occurred before the radicle emergence in the seed. All these metabolic alterations halt the seed imbibition and lowered seed moisture to its initial level. Thus, seed again imbibes water and triggers the process of cell division and elongation along with ATP production that allow the radicle emergence [33–35]. Previous studies suggested that crops raised from primed seeds mature earlier with improved thermotolerance and seed priming could be an effective strategy for sustainable yield [36–38]. A recent study also suggested that exogenous application of different growth regulating compounds (hormones, antioxidants, micro or macronutrients, and nanoparticles) significantly improve the plant growth by alleviating the damaging effects of abiotic stresses. These exogenously applied ameliorants positively regulates the synthesis and functional activities of osmo-protectants, enzymatic and non-enzymatic antioxidant related defensive compounds which leads to sustainable plant productivity [39]. Particularly, seed priming with calcium chloride (CaCl_2_) or potassium nitrate (KNO_3_) breaks the high temperature stress induced seed dormancy and promote the uniformed seed germination with increased plant density and biomass accumulation [40]. Previous studies reported that seed priming with KNO_3_ increased the germination of mountain ash, cabbage, rice, cantaloupe, cotton, rice, melon, tomato under non-stressed and stressed conditions but this effect vary among the species and environmental conditions [41–46]. No study has yet been reported regarding effects of seed priming with KNO_3_ on carrot crop despite being a profitable commodity for the farmers (Supplementary file Table 1) in the Punjab province (contributes 80% of total country’s production) of Pakistan [47]. Carrot is a winter crop and typically sown in late September, farmers often choose to sow it in late August to maximize profits as an early crop. In an attempt to achieve an optimal plant population, farmers employ a higher seed rate, usually twice the normal rate. Unfortunately, poor seed germination remain a persistent issue due to high temperatures during this period. Seed priming with potassium nitrate presents a potential solution to enhance carrot resilience under high temperature conditions as seed germination is the most disturbed event. Understanding the specific mechanisms and effects of KNO_3_ seed priming on mitigating high temperature stress in carrots is essential for optimizing cultivation strategies in the current prevailing environmental conditions. Keeping in view the above scenario, the present study was conducted (1) to measure the efficacy of seed priming with different treatments of KNO_3_ in terms of in vitro seed germination and seedling growth parameters under high temperature stress compared to non-primed seeds, (2) to identify the optimum level of KNO_3_ for carrot seed priming at seed germination and subsequent seedling growth under high temperature stress in in vitro conditions, (3) to assess the efficacy of optimized seed priming treatments in terms of seedling emergence and growth, physiological and biochemical responses of KNO_3_-primed carrot plants to high temperature stress, focusing on antioxidant activity, osmotic adjustment, and membrane stability in open field conditions.

## Materials and methods

### In vitro preliminary experiment

Seeds of a local carrot cultivar ‘T-29’ were obtained from Vegetable Seed Lab. (VSL), University of Agriculture, Faisalabad, Pakistan. Initially carrot seed moisture content was measured with seed moisture meter (GMK 503 A) and moisture contents for hundreds of seeds were ranged from 8 to 8.5%. These seeds were used for primed with various aerating solutions of KNO_3_. Briefly, seeds without priming were taken as negative control (T_0_: no priming), while seeds primed with water were taken as positive control (T_1_: hydro-priming) and seeds treated with KNO_3_ solutions as nutrient-priming (T_2_: 50 mM, T_3_: 100 mM, T_4_: 150 mM, T_5_: 200 mM, T_6_: 250 mM, and T_7_: 300 mM). Selection of KNO_3_ concentration was based on previous study [48, 49]. In detail, applied concentrations of KNO_3_ were prepared by dissolving the potassium nitrate salt in deionized water using magnetic stirrer and hot plate (BTI-27) for 15 min. After that, seeds were soaked in KNO_3_ solutions by keeping seed to solution constant ratio of 1:5 (g/ml) in complete darkness at room temperature with continuous-flow aeration for 12 h [50]. Later, primed seeds were air-dried at room temperature until seeds reach their original moisture level. The primed air-dried seeds were re-hydrated on moisturized filter paper in petri dishes. Total of 20 seeds were placed in each per dish from all treatments in four replicates and petri dishes were incubated at 35 ± 2℃ in incubator for ten days. Selection of temperature was based on earlier study [6, 51] and seeds with 1 mm radicle protrusion were counted as germinated and observations were recorded after two days interval. After ten days of incubation, five healthy seedlings were taken from each treatment and were subjected to various measurements to determine the optimized level of KNO_3_ for better seedling growth under high temperature stress. Seedling length was measured with measuring tape in cm and seedling fresh weight was measured with digital electronic balance in mg. seedlings were oven dried at 65℃ for three days and were weighed for dry biomass. Seedling vigor index was determined as computed in a previous study [52]. Final seed germination percentage, time to 50% germination, mean germination time, germination energy, and germination index were calculated to determine the optimized level of KNO_3_ for seed priming to enhance the high temperature stress tolerance in carrot as previously reported by Ziaf et al. [53].

Recorded data was subjected to analysis of variance (ANOVA) and mean comparison analysis to optimize the effective dose of KNO_3_ for seed priming on the basis of significant variation (*p*-value < 0.0001) for amelioration of high temperature stress in carrot at seed germination and subsequent growth stages.

### Field experiment

The efficacy of optimized level of KNO_3_ for seed priming in mitigating high temperature stress in carrots at seedling emergence and subsequent growth stage was assessed in open field condition. For this, a two-year field experiment was conducted at Vegetable Research Area (VRA), University of Agriculture, Faisalabad, Pakistan. Experiment was arranged in randomized complete block design (RCBD) in three replications. Carrot seeds were hydro-primed (positive control) and primed with two optimized treatments (exhibited significant variation at *p*-value < 0.0001) of KNO_3_ (50 mM and 150 mM) selected from in vitro preliminary experiment for 12 h.

### Growth conditions

After completion of priming process, seeds were planted at seeding rate of 8 kg/acre directly in the field on well-prepared beds (2.5ft$\times$ 10ft) during August for both years on both sides of the beds. Meteorological data of the experimental location is presented in Table 1. Recommended fertilizers (50:50:50 kg acre^− 1^) were added to the soil in form of urea (three splits) while, phosphorus and potash were applied as a basal dose before bed formation. First irrigation was provided immediately after seeding and further filed was irrigated as per crop requirement. Crop thinning was done after 2nd week of sowing while, hoeing and weeding performed accordingly with other standard crop management practices.

**Table 1:** Metrological data of experimental location during crop cycle of carrot

Year 1	Temperature (℃) (Average)			*R*.H (Ave.)	Rain fall (Ave.)	Sunshine (Ave.)	Wind speed (Ave.)
	Maximum	Minimum	Average	(%)	(mm)	(Hours)	km/h
Aug	35.9	26.7	31.3	60.4	48.4	7	4.3
Sep	35.4	24.4	29.9	51.6	75.2	8.2	3.6
Oct	32.2	19.1	25.4	52.9	14.5	7	3.6
Nov	27.1	12.1	19.6	61.5	8.8	6.6	2.6
**Year 2**							
Aug	35.7	26.5	31.1	62.2	48.1	7	4.2
Sep	36.5	25.5	31	53.6	12	7.8	3.5
Oct	33.9	19.6	26.7	51.3	22.2	7	3.6
Nov	27.6	12.6	20.1	60.1	0.00	6.4	2.6

*Source* AgroMet observatory, Dep. of Agronomy, UAF

### Data collection

Data was recorded during the seedling stage (10 days post-sowing) and at the harvesting stage of the carrot crop (90 days after sowing). Number of emerged seedlings on both sides of beds was counted after 10 days of sowing. Final seedling emergence percentage and seedling vigor index were calculated method described by [4, 52].

### Enzyme and related activities

During field evaluation at seedling stage, 1.0 gram of fresh leaf samples from each treatment were homogenized in 2.0 mL of phosphate buffer (pH: 7.2) and homogenate was centrifuged at 10,000 rpm for 10 min. Supernatant was used to assess the various stress responsive enzymatic and antioxidant activities.

#### Estimation of peroxidase (POD)

Activity of POD was estimated by following a method used by [54]. For this estimation, 0.2 mL of plant supernatant was mixed with phosphate buffer (2.5 mL), 1% guaiacol solution (0.2 mL), and 0.3% H_2_O_2_ solution (0.3 mL). Absorbance was measured at 470 nm spectrophotometrically and activity was expressed in U Kg^− 1^ of total soluble proteins.

#### Estimation of catalase (CAT)

Activity of CAT was measured by following method reported by [55], and later on modified by [54]. For this estimation, 0.2 mL of plant supernatant was mixed with 25 mM of phosphate buffer of pH 7.0 and 10 mM of freshly prepared hydrogen peroxide. Catalase activity was calculated by degradation of 1.0 mol of H_2_O_2_ min^-1^ mg^-1^ of protein and was expressed in U µmol g ^-1^ of fresh weight of plant tissues.

#### Estimation of superoxide-dismutase (SOD)

Activity of stress responsive SOD enzyme was measured by the method of [56] and later on modified by [57]. For this estimation, 0.1 mL of plant supernatant was mixed with 130 mM of methionine, 1mM of EDTA, 0.75 mM of NBT, 0.02 mM riboflavin, and 50 mM of phosphate buffer. Absorbance was measured at 560 nm spectrophotometerically after 7 min exposing the reaction mixtures to fluorescent and activity of SOD was expressed in U Kg^-1^ of total soluble proteins.

#### Total phenolic and antioxidant contents (%)

Total phenolic contents were measured according to the method described by [58] spectrophometrically using gallic acid as internal standard. Similarly, total antioxidant contents were measured by following method described by [59] using DPPH assay.

#### Malondialdehyde contents (µmol/g FW seed)

A method reported by [60] was used to estimate the malondialdehyde contents in carrot seedlings to determine the lipid peroxidation in response to high temperature stress.

#### Photosynthetic characteristics

Three mature leaves per plant of four weeks old seedlings were selected and placed in the chamber of a portable Infra-Red Gas Analyzer (LCi-SD, ADC Bio-scientific, UK) one by one. Data was recorded for photosynthetic rate (µmol m^2^ s^− 1^), transpiration rate (mmol m^− 2^ s^− 1^), and stomatal conductance [61]. Additionally, water use efficiency was calculated by taking ratio of photosynthetic rate to transpiration rate and was expressed in pmol CO_2_ mmol^− 1^ H_2_O.

#### Carrot yield and yield contributing root traits

At the harvesting, plants were uprooted, washed and yield contributing root traits were measured for each treatment. Root length and root weight was measured for five plants from each replicate of all treatment and mean values were expressed in cm and g respectively. Root yield (RY) was assessed by harvesting carrot roots from small beds having area of 12.5 ft^2^ for each replication of a treatment.

### Statistical analysis

The recorded data was subjected to analysis of variance, and the mean comparison analysis of all treatments using the Highest Significant Differences (HSD) test using R.studio version 3.2.4. Correlation, principal component analysis (PCA), and heat maps were also drawn for better visualization of data.

## Results

### Electrical conductivity

A preliminary experiment was conducted to optimize the level of KNO_3_ for seed priming to boost seed germination potential under high temperature stress. Electrical conductivity of primed and unprimed seeds was measured at 1 h, 3 h, 6 h, 12 h, and 24 h after soaking in different concentrations of KNO_3_ ranging from 50 mM to 300 mM along with hydro-priming (positive control) and no priming (negative control). Maximum EC values were recorded for unprimed seeds followed by seeds primed with higher concentrations of KNO_3_ (Supplementary file Table 2). Low EC values were recorded for seeds primed with distilled water and low concentration of KNO_3_ (50 mM, and 150 mM). Additionally, it has been noticed that EC values of primed seeds were negatively related with time for seed soaking in solutions for all the treatments. Thus, efflux of ions was reduced in seeds primed for 12 and 24 h and initial radicle emergence was increased in seeds having low ions efflux.

### Seed germination essay

**Fig. 1:**
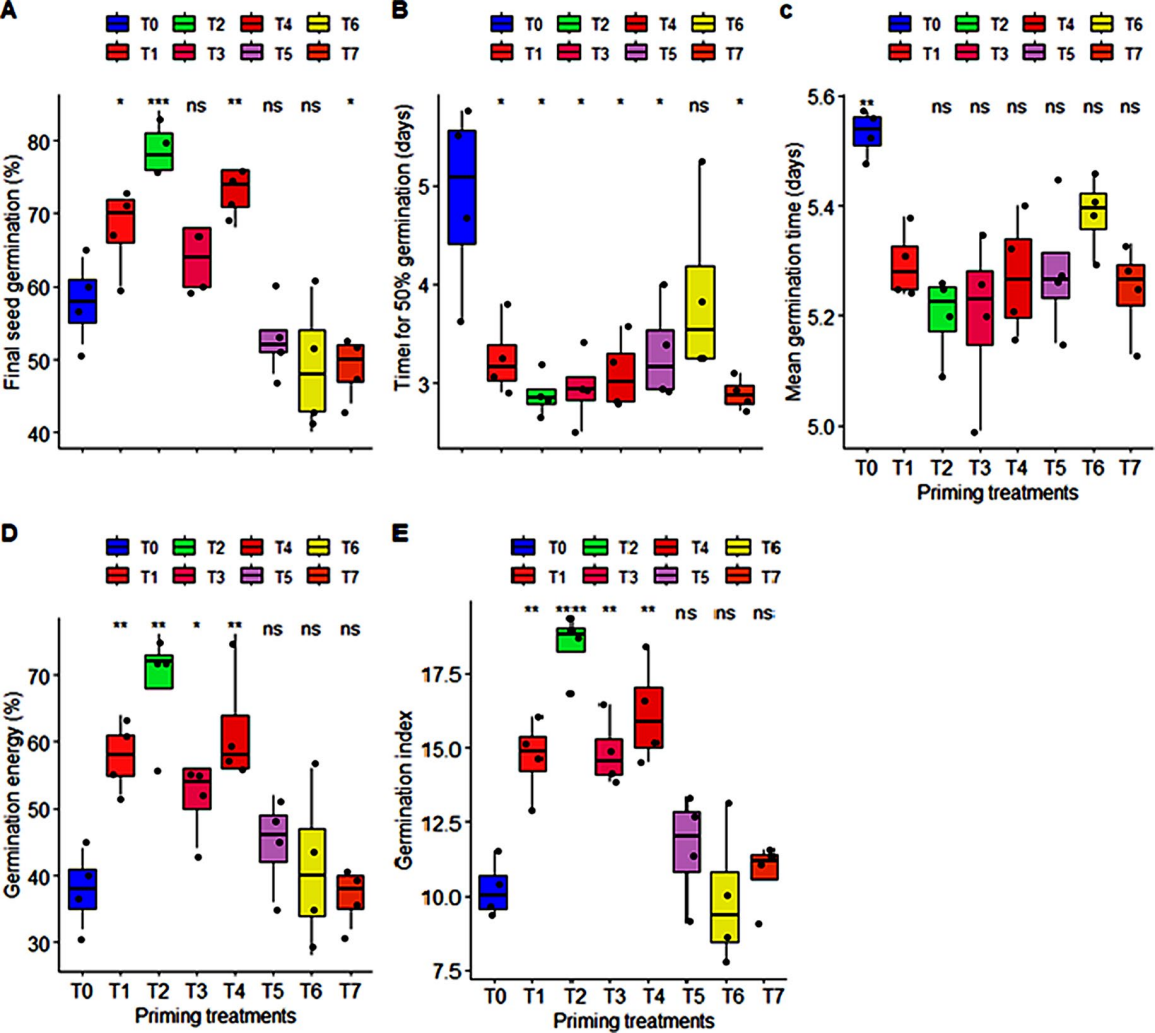
Classified filled boxplots of (**A**) final seed germination (%), (**B**), time taken for 50% germination in days, (**C**) mean germination time in days, (**D**) germination energy (%), and (**E**) germination index of a carrot cultivar T-29. The eight priming treatments of KNO_3_ from T0 to T7 represented by different color filled boxplots. Each boxplot is delimited 25th and 75th percentile with error bars represents four replicates from each treatment and include a solid line represents the mean value. One way ANOVA calculated significant differences among the treatment represented with *** (*P* < 0.0001), ** (*P* < 0.001), * (*P* < 0.05), and ns: non-significant (*P* > 0.05) notations

Potassium nitrate (KNO_3_) seed priming positively affected the germination attributes of carrot seeds including final seed germination (%), time for 50% seed germination, mean germination time, germination energy, and germination index in comparison to both positive (hydro-priming) and negative (no priming) control at 35 ± 2^°^C. Analysis of variance exhibited that effect of T2 (50 mM of KNO_3_), T4 (150 mM of KNO_3_) were highly significant (*P* < 0.0001, and *P* < 0.001 respectively) on final seed germination (%) among all priming treatments. Maximum seed germination (79%) was recorded in seeds primed with 50 mM of KNO_3_ which was statistically at par with seeds primed with 150 mM of KNO_3_ with 75% seed germination. Additionally, T1, T2, T3, and T4 significantly enhanced the germination energy and germination index of carrot seeds whereas; maximum variation in germination energy and germination index was calculated for T2 under high temperature stress. Furthermore, it is obvious from analysis of variance that maximum time of 4.89 days and 5.53 days was taken for 50% seed germination and mean germination by unprimed seeds followed by seeds primed with higher concentration (250 mM, and 300 mM) of KNO_3_ and minimum time of 2.88 days and 5.2 days was taken by seeds primed with 50 mM of KNO_3_ (T2) respectively but these responses did not significantly varied among different priming treatments under high temperature stress (Fig. 1). Thus, present study suggests that seed priming with 50 mM and 150 mM of KNO_3_ can be useful to enhance the rate and speed of carrot seeds under high temperature stress conditions.

### Seedling establishment

**Fig. 2:**
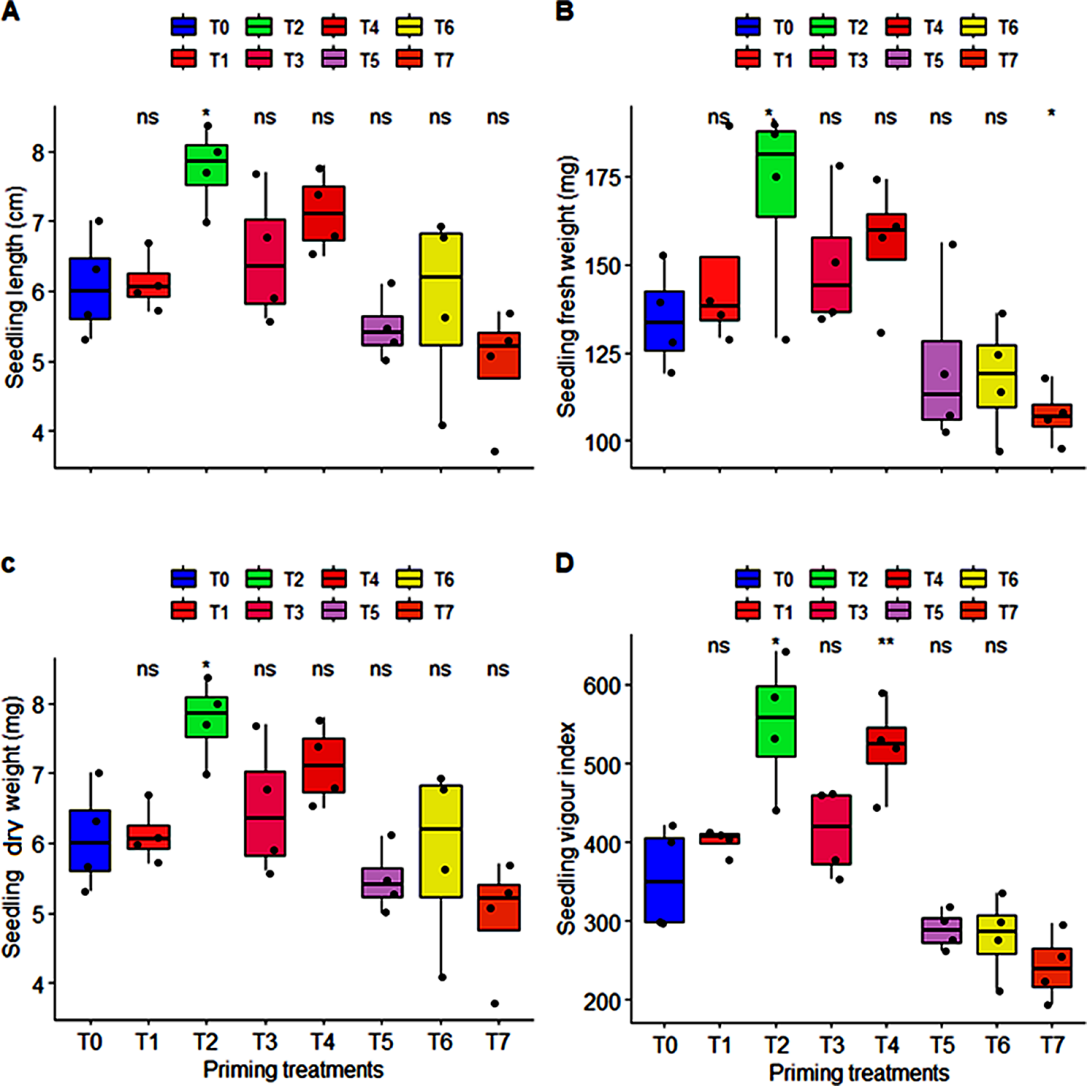
Classified filled boxplots of (**A**) seedling length (cm), (**B**), seedling fresh weight (mg), (**C**) seedling dry weight (mg), (**D**) seedling vigour index of carrot seedling. The eight priming treatments of KNO_3_ from T0 to T7 represented by different color filled boxplots. Each boxplot is delimited 25th and 75th percentile with error bars represents four replicates from each treatment and include a solid line represents the mean value. One way ANOVA calculated significant differences among the treatment represented with *** (*P* < 0.0001), ** (*P* < 0.001), * (*P* < 0.05), and ns: non-significant (*P* > 0.05) notations

Seedling establishment potential of KNO_3_ primed germinated seeds was evaluated at 35 ± 2^°^C under in vitro conditions. Analysis of variance exhibited that effect of all priming treatments (except T2: 50 mM of KNO_3_) was non-significant on seedling establishment attributes (seedling length, seedling fresh weight, seedling dry weight, and seedling vigor index) under high temperature stress conditions. Maximum seedling length of 7.78 cm with highest value of fresh weight (176 mg), and dry weight (7.89 mg) was recorded for seedling emerged from seeds primed with lower concentration of KNO_3_ (T2: 50 mM) and these measurements were found comparably close with 150 mM of KNO_3_ (T4) under high temperature stress condition, and seedling vigor index values were also high (549, and 521 respectively) as compared to seedlings emerged from other unprimed and primed seeds. On the other hand, lowest measurements for all studied attributes of seedling establishment were recorded for the seedlings emerged from seeds primed with higher concentration of KNO_3_ (T6: 250 mM, and T7: 300 mM) (Fig. 2).

### Multivariate analysis of data to evaluate the efficacy of seed priming

**Fig. 3:**
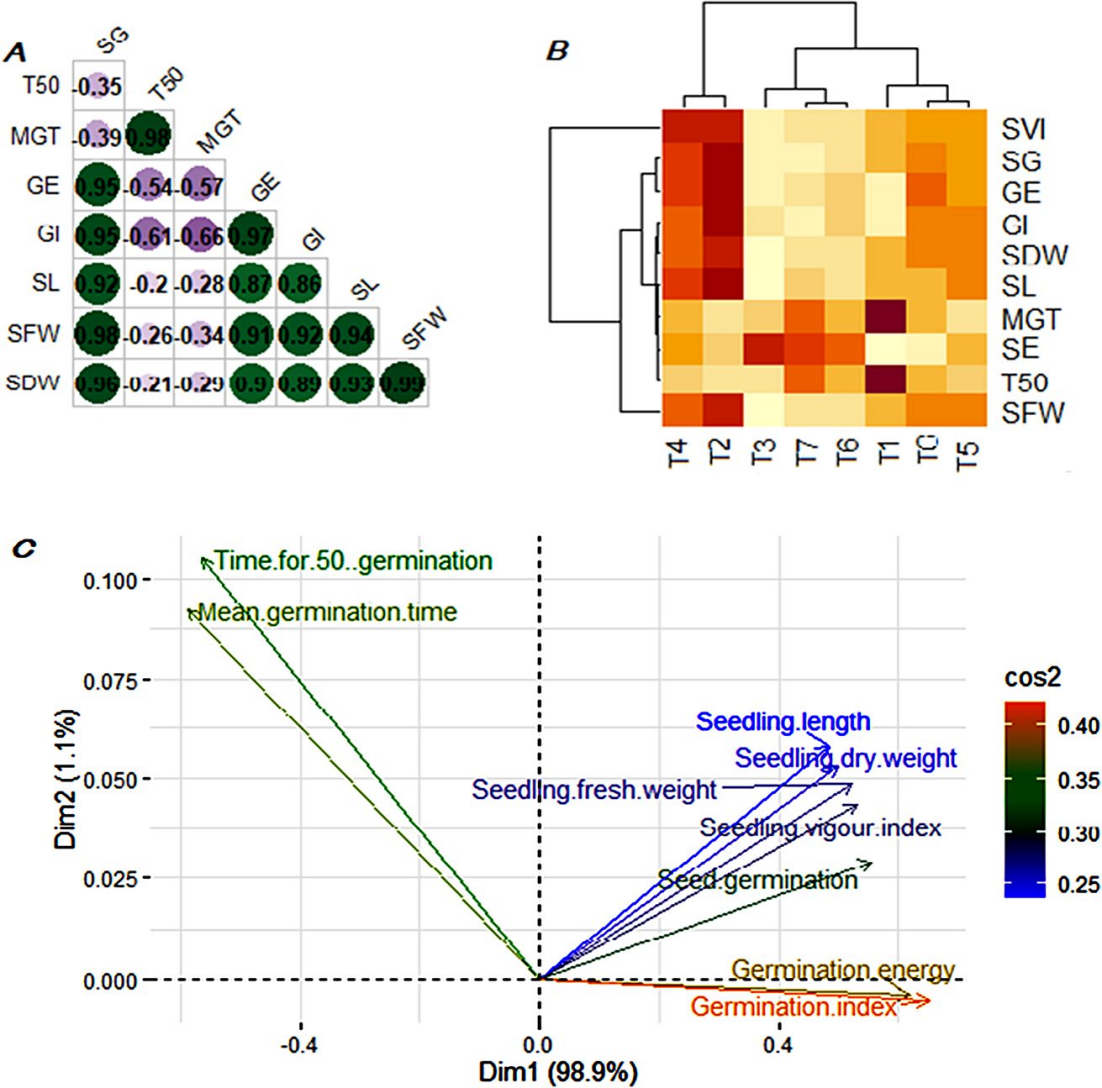
**a.b.c** Multivariate analysis of preliminary experiment (**A**) pearson’s correlation coefficients among pairs of response variables of carrot cultivar, (**B**) cluster heatmap of eight priming treatments and 10 response variables of seed germination and seedling establishment, (**C**) biplot of principal components of response variables. Purple dots represents negative while dark green dots represent positive correlation coefficients between response variables. Color intensity and size of dots represents the strength of dependency between pairs of response variable. Euclidian distance-based clustering in the heatmap exhibit three separate clusters for priming treatments and four separate clusters for response variables. All response variables were plotted in three directions in principal component based biplot

Pearson’s correlation coefficients among ten germination and seedling establishment attributes of KNO_3_ primed seeds have been computed and presented in Fig. 3a. The correlation coefficients were tested for significance at 5% confidence level. Under in vitro conditions, seed germination was found in strong positive association (*r* > 0.90 each) with germination energy and germination index, and seedling establishment attributes whereas, time for 50% germination and mean germination time exhibited a negative association with all other studied attributes of seed germination and seedling establishment (*r* = -0.20 to -0.61). The hierarchical clustering based heatmap of 10 studied growth attributes of primed seeds and 8 priming treatments was presented in Fig. 3b. Euclidian distance based clustering exhibited three separate clusters for priming treatments where T2 and T4 fall in one group and the remaining treatments fall in another group with 2 sub-groups whereas, response variable formed four separate clusters. Additionally, principal component analysis (PCA) a dimensionality reduction technique has been used to categorize the growth attributes in terms of principal components (PCs) and presented in Fig. 3c. In this study, it was found that first two PCs explained 99.9% of the variability in the data set. Important variables have been identified on the basis of higher vector loadings among four PCs. Seed germination, seedling length, seedling fresh weight, seedling dry weight, seedling vigor index majorly contributed to PC1 whereas, germination energy and germination index also contributed to PC1. Loadings of time for 50% germination, and mean germination time majorly contributed to PC2.

### Role of optimized priming treatments (H_2_O and KNO_3_) on seedling and physiological performance of carrot under field conditions

**Fig. 4:**
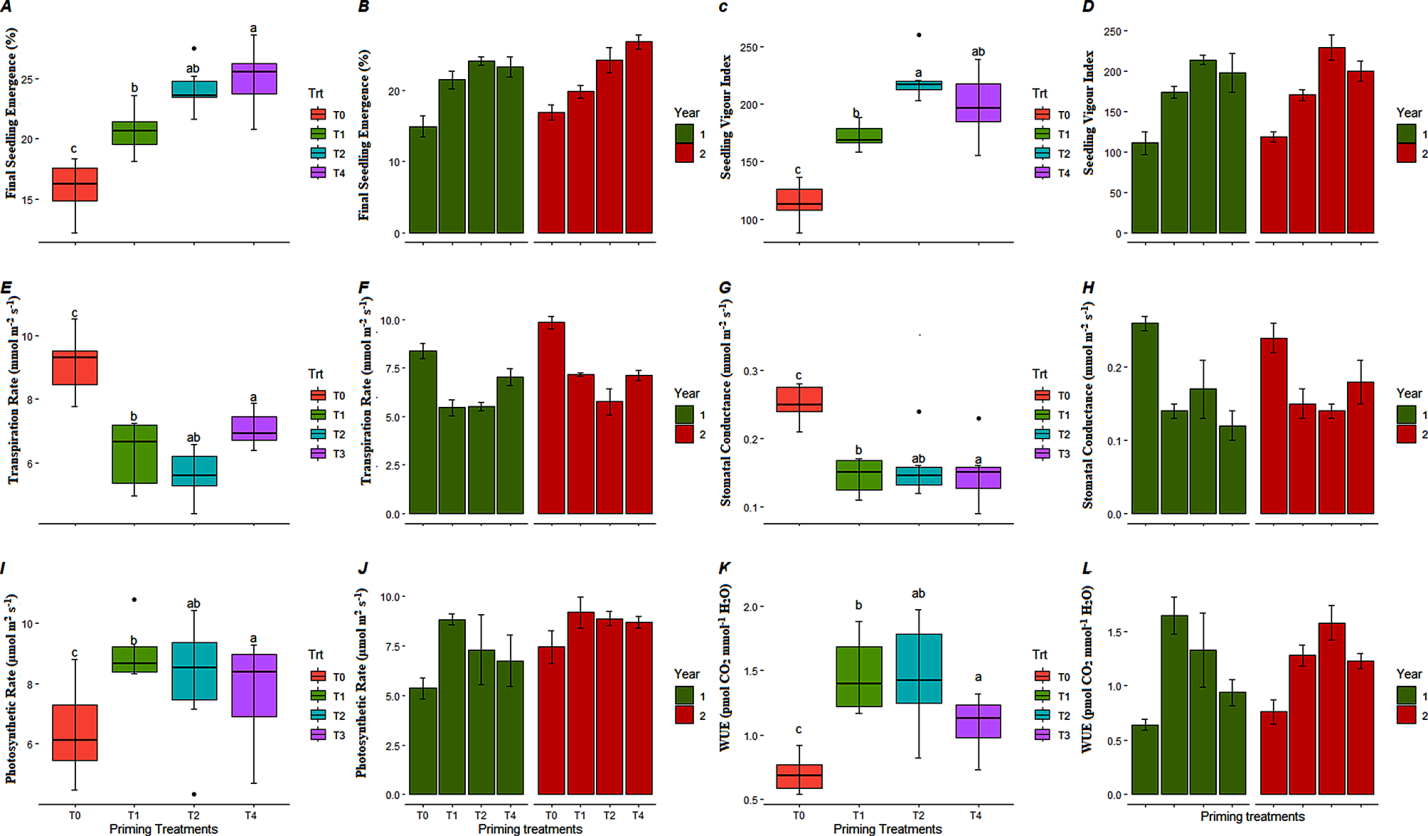
Classified filled boxplots and barplots of (A, B) final seed emergence, (C, D), seedling vigour index, (E, F) transpiration rate, (G, H) stomatal conductance, (I, J) photosynthetic rate, (K, L) water use efficiency of carrot seedlings respectively. In boxplots, four optimized priming treatments (T0, T1, T2, and T4) of KNO_3_ represented by different colors. Each boxplot is delimited 25th and 75th percentile with error bars represents four replicates from each treatment and include a solid line represents the mean value. Two way ANOVA (treatments and years) calculated significant differences, followed by LSD test based lettering are also presented, where same letter did not vary significantly. In barplots, two years represented by different colors, each barplot is presenting mean value and error bar represents standard error of four replicates

Two optimum osmoprimng treatments of KNO_3_ (T2: 50 mM, and T4: 150 mM) along with positive (hydropriming), and negative (no priming) control were evaluated under open field conditions. Final seedling emergence, seedling vigor index, and physiological attributes such as transpiration rate, stomatal conductance, photosynthetic rate, and water use efficiency of carrot seedlings were studied under high temperature in open field conditions for two consecutive years. Two factor (priming treatments, and year) analysis of variance followed by LSD test at 5% level of significance has been calculated and presented in Fig. 4. The findings exhibited that final seedling emergence (%), and seedling vigor index significantly varied from 14.8 to 26.7%, and 110 to 288 units in response to different priming treatments respectively. It was observed that maximum seedling emergence in both years was recorded in response of seed priming with 50 mM and 150 mM of KNO_3_ as compared to control conditions. Additionally, maximum seedling vigor index was calculated for seedlings emerged from seeds primed with 50 mM of KNO_3_ in both years i.e. 213 and 228 respectively (Fig. 4a-d).

Moreover, all physiological attributes transpiration rate, stomatal conductance, photosynthetic rate, and water use efficiency in carrot seedling also significantly varied in response of different priming treatments. Transpiration rate and stomatal conductance were decreased in seedling emerged from hydro-primed and 50 mM KNO_3_-primed seeds (~ 5 mg H_2_O m^− 2^s^− 1^, 0.12 to 0.14 mmol m^− 2^s^− 1^ respectively) as compared to unprimed seeds. Similarly, photosynthetic activity and water use efficiency was higher in seedlings emerged from 50 mM KNO_3_-primed seeds (9.19 µmol m^− 2^s^− 1^, and 1.65 pmol CO_2_ mmol^− 1^ of H_2_O respectively) followed by hydro-primed seeds in both years (Fig. 4e-l).

### Stress responsive antioxidant defense system

**Fig. 5:**
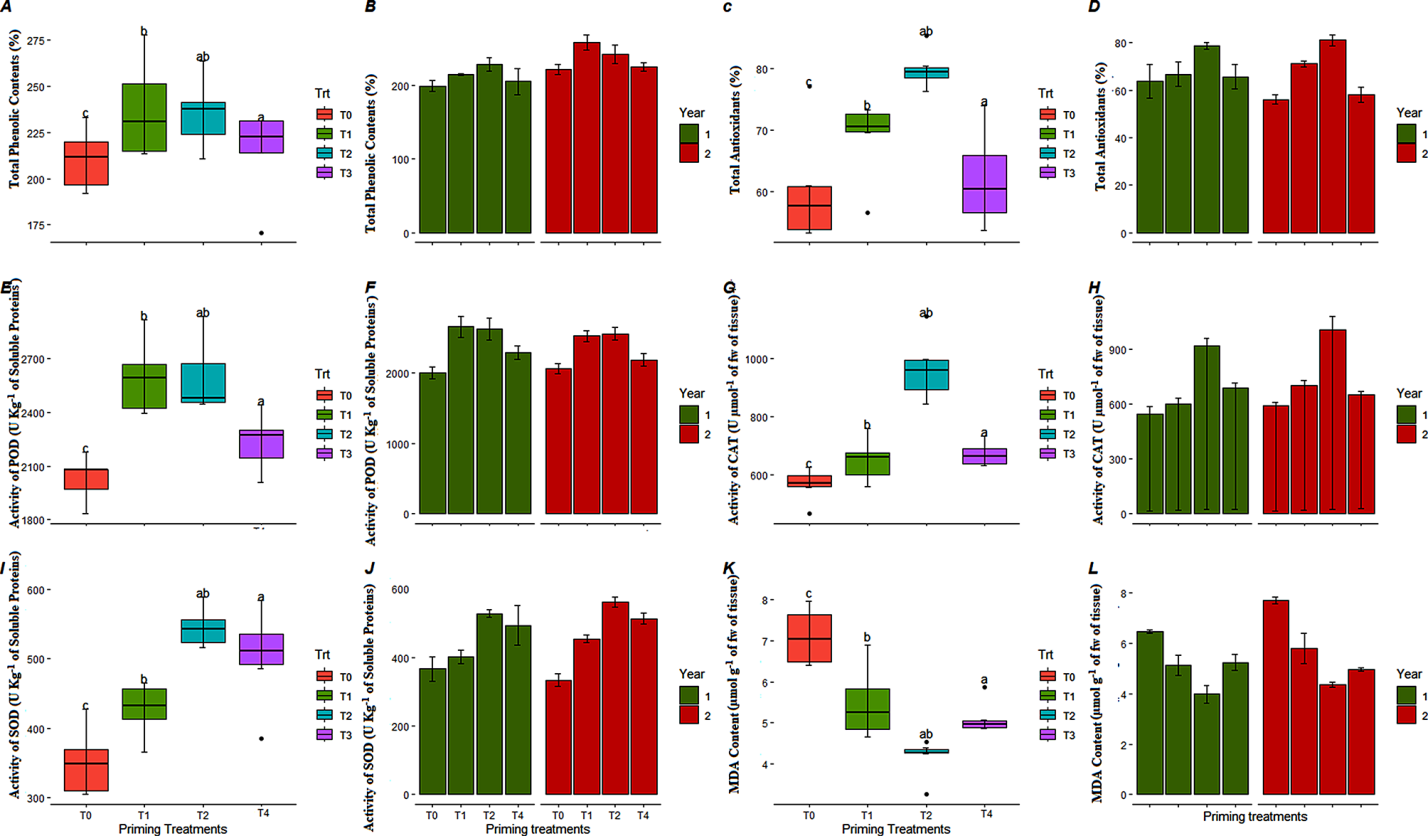
Classified filled boxplots and barplots of (**A**, **B**) total phenolic contents, (**C**, **D**) total antioxidants, (**E**, **F**) activity of POD, (**G**, **H**) activity of CAT, (**I**, **J**) activity of SOD, (**K**, **L**) activity of MDA in carrot leaves respectively. In boxplots, four optimized priming treatments (T0, T1, T2, and T4) of KNO_3_ represented by different colors. Each boxplot is delimited 25th and 75th percentile with error bars represents four replicates from each treatment and include a solid line represents the mean value. Two way ANOVA (treatments and years) calculated significant differences, followed by LSD test based lettering are also presented, where same letter did not vary significantly. In barplots, two years represented by different colors, each barplot is presenting mean value and error bar represents standard error of four replicates

Analysis of variance followed by LSD test showed that total phenol contents (TPC) and total antioxidants (TA) in leaves of carrot seedlings (grown under high temperature stress) varied from 199 to 258 units g-1 of fresh weight of plant tissues under different priming treatments (Fig. 5a-d). Results showed that TPC increased in response to KNO_3_ priming treatment (T2) up to 241 and 258 units over unprimed treatment in both years respectively (Fig. 5b). Similar trend was observed for total antioxidants in both years. Moreover, activities of stress responsive enzymes such as catalase, peroxidase, and superoxide dismutase were also increased in leaves of plants emerged from primed seeds as compared to unprimed seeds. Highest activity of peroxidase (2659, and 2622 U kg^− 1^ of total soluble proteins), catalase (919.3, and 1006.7 U µmol g^− 1^ of tissues), and superoxide dismutase (528.1, and 561.7 U kg^− 1^ of total soluble proteins) was measured in leave plant tissues in response to 50 mM KNO_3_ priming among all priming treatments in both years respectively (Fig. 5e-j). Additionally, malondialdehydes contents were decreased in plant leaves in response of priming treatments over plants emerged from unprimed seeds. Highest membrane degradation was measured in terms of lipid peroxidation by activity of MDA in leaves of plants emerged from unprimed seeds in response to heat stress conditions (Fig. 5k-l). Thus, present study suggests that seed priming has improved the defense mechanism of the seedling by enhancing the enzyme activities.

### Carrot yield contributing root traits

**Fig. 6:**
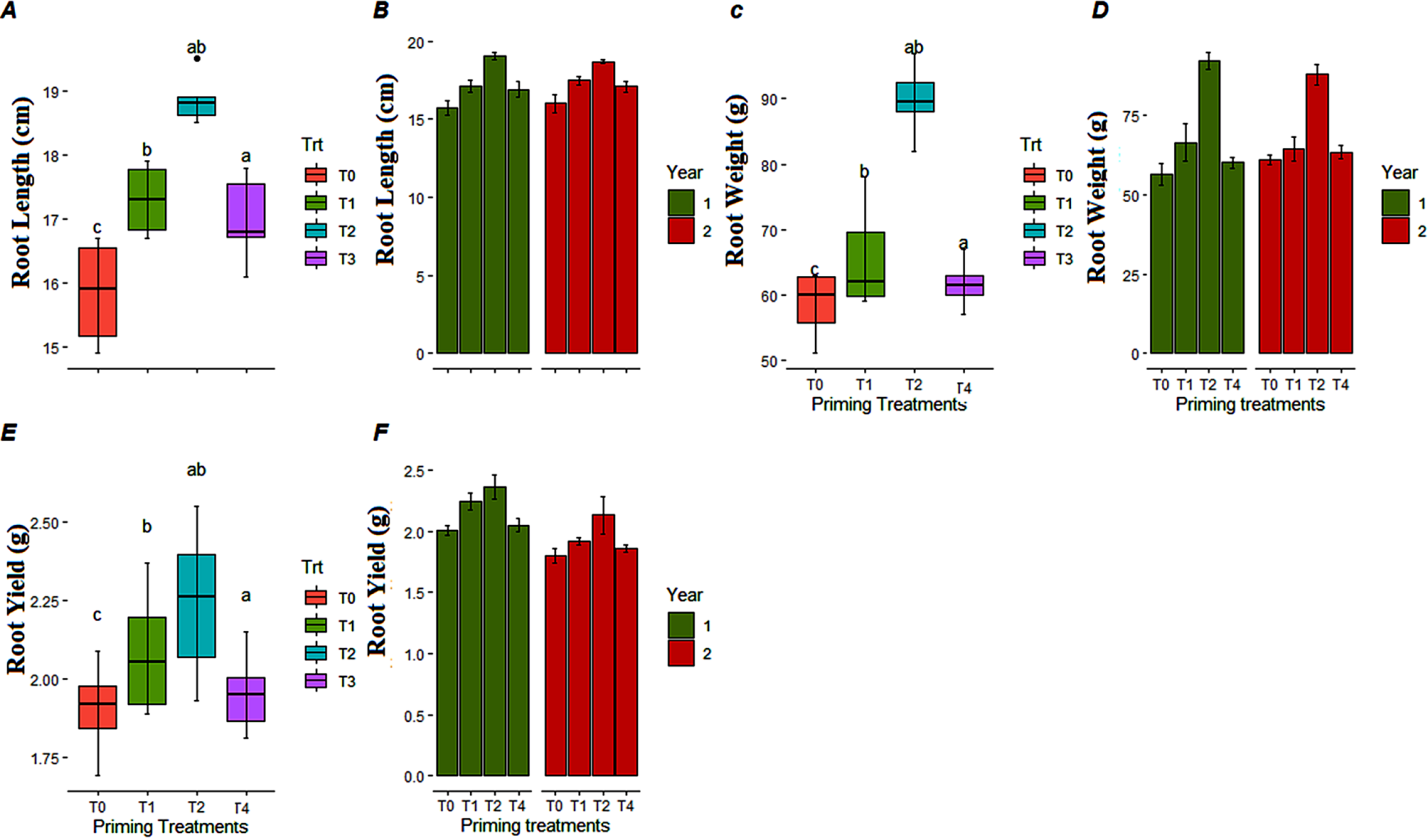
Classified filled boxplots and barplots of (**A**, **B**) root length, (**C**, **D**) root weight, (**E**, **F**) carrot root yield of carrot plants respectively. In boxplots, four optimized priming treatments (T0, T1, T2, and T4) of KNO_3_ represented by different colors. Each boxplot is delimited 25th and 75th percentile with error bars represents four replicates from each treatment and include a solid line represents the mean value. Two way ANOVA (treatments and years) calculated significant differences, followed by LSD test based lettering are also presented, where same letter did not vary significantly. In barplots, two years represented by different colors, each barplot is presenting mean value and error bar represents standard error of four replicates

For yield and yield components, significant differences were observed among different priming treatments. Maximum root length, root weight and carrot root yield were measured in plants emerged from seeds primed with 50 mM KNO_3_ followed by 150 mM of KNO_3_ which was statistically at par with hydro-priming in both years (Fig. 6). This study suggests that seed priming with lower concentration of KNO_3_ could be a useful approach to enhance the carrot root yield by avoiding the adverse effects of high temperature stress at seed germination and seedling establishment stage.

### Multivariate analysis of data to evaluate the efficacy of of growth and yield

**Fig. 7:**
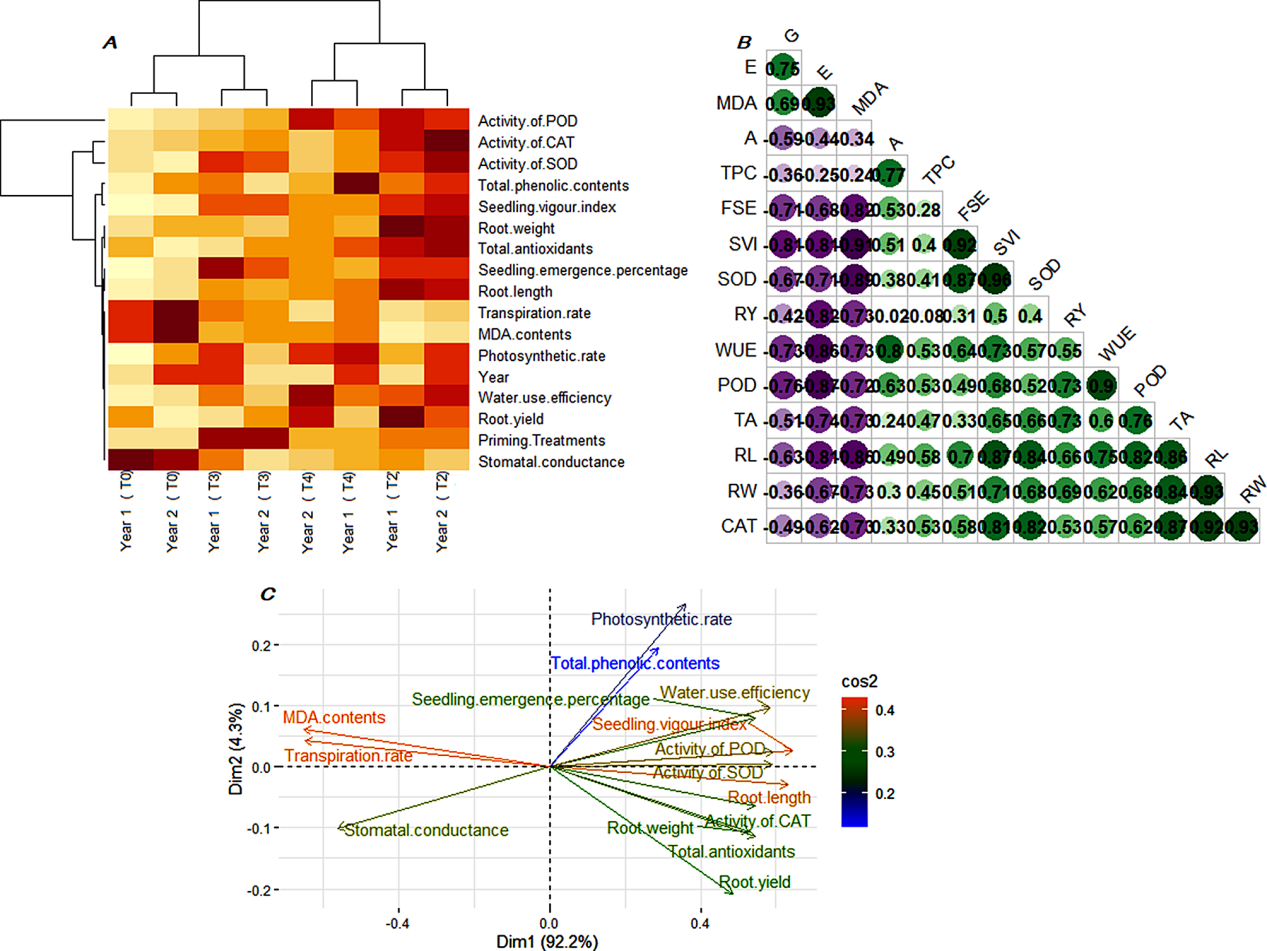
Multivariate analysis of preliminary experiment (**A**) cluster heatmap of four optimized priming treatments and 15 response variables of carrot, (**B**) pearson’s correlation coefficients among pairs of response variables of carrot cultivar, (**C**) biplot of principal components of response variables. Purple dots represents negative while dark green dots represents positive correlation coefficients between response variables. Color intensity and size of dots represents the strength of dependency between pairs of response variable. Euclidian distance based clustering in the heatmap exhibit three separate clusters for priming treatments and four separate clusters for response variables. All response variables were plotted in three directions in principal component based biplot

The hierarchical clustering based heatmap of 15 studied growth and yield attributes of primed seeds and 4 selected priming treatments was presented in Fig. 7a. Euclidian distance based clustering exhibited two homogenous clusters for priming treatments where T2 and T4 fall in one group and the remaining treatments fall in another group with 2 in both years whereas, response variable formed four separate clusters. Pearson’s correlation coefficients among fifteen growth and yield attributes of plants emerged from KNO_3_ primed seeds have been computed and presented in Fig. 7b. The correlation coefficients were tested for significance at 5% confidence level. Under open field conditions, all studied plant growth and yield traits were found in strong positively association with each other except stomatal conductance and transpiration rate. Additionally, principal component analysis based categorization the growth and yield attributes was presented in Fig. 7c. In this study, it was found that first two PCs explained 96.5% of the variability in the data set. Seedling emergence, seedling vigor index, water use efficiency, activity of POD and SOD fall in one group along with total phenolic contents and photosynthetic rate majorly contributed to PC1. Whereas, root and root components (root length and root weight) along with activity of catalase also contributed in variability depicted by PC1. Loadings of MDA content and transpiration were closely associated with stomatal conductance and they majorly contributed to PC2.

## Discussion

Potassium is not only an essential nutrient for plant growth also mitigates the adverse impacts of high temperature stress by modulation of photosynthesis, translocation of water-soluble carbohydrates, and antioxidant defense mechanism [62]. In present study, seed priming with potassium nitrate improved the high temperature stress tolerance in carrot under field and in vitro conditions. It was observed that final germination percentage was improved maximum by priming with 50 mM of KNO_3_ while high concentrations of KNO_3_ (200–300 mM KNO_3_) didn’t succeeded in improving the germination potential or seedling growth characteristics of carrots. The unprimed seeds exhibited the highest T_50_ value whereas seeds primed with 50 mM KNO_3_ achieved 50% germination in the shortest time. KNO_3_ regulates water uptake and maintain seed moisture level which is crucial for initiation of several metabolic processes to break the seed dormancy even in drought or elevated temperatures [63, 64]. Nitrate ions induce an osmotic potential that facilitates the more water imbibition in primed seeds [65]. Furthermore, seed priming with KNO_3_ facilitate the repair of deteriorated tissues such as cellular membranes, DNA and stress responsive proteins in abiotic stressed seeds by scavenging the reactive oxygen species [66] it has also been evidenced in the present study that ion outflow was decreased in primed seeds in response of repaired cellular membranes. Previous studies reported similar effects of seed priming with low concentration of KNO_3_ in tomato and sorghum, under abiotic stress conditions [67, 68]. Low concentration of KNO_3_ maintains the level of nitrate ions and facilitates the plant to balance the other essential nutrients, osmolytes required for growth and protect the oxidative stress induced damages [69–71]. Additionally, low concentration of KNO_3_ assists the activity of amylase and protease enzymes which solubilize and mobilize the seed reserves for embryonic growth by providing energy during germination and boost the seedling vigor to withstand under high temperature stress [44, 63, 72, 73]. Similar findings were reported in eggplant and maize hybrids [74–77]. These findings suggest that seed priming with KNO_3_ is an effective strategy with considerable economic benefits for farmers [78, 79].

Generally, high temperature stress induces the oxidative damages by overproduction of reactive oxygen species which cause harm to DNA, protein and lipid molecules and indicated by increased production of MDA contents in stressed plants [80]. In response of these metabolic alterations, plants activate production of stress responsive enzymatic (SOD, POD, CAT) and non-enzymatic (phenolics, falavonoids) antioxidant contents [81]. Increased production and activities of these antioxidants also triggers the more accumulation of several osmoprotective metabolites such soluble sugars, free amino acids to maintain the osmotic potential and turgor potential necessary for the water uptake to prevent the tissue desiccation [82]. As in the present study, it was observed that level of stress responsive enzymatic and non-enzymatic antioxidants was decreased in plants raised from unprimed and hydro-primed seeds under high temperature stress conditions. However, seed priming with KNO_3_ increased the antioxidant contents (total phenolic, MDA, and total antioxidants) and enzymatic activities of SOD, POD, and CAT in carrot plants under high temperature stress conditions. Seed priming with KNO_3_ played a significant role in alleviation of high temperature stress by improving the stress tolerance level in plants as compared to plants raised from unprimed and hydro-primed seeds. Thus present study also suggest that ion outflow, overproduction of ROS, membrane lipid peroxidation, antioxidant production, stress responsive enzymes activity, induced thermotolerance are interlinked with regulation of carrot seedling responses towards stress by seed priming KNO_3_. Additionally, non-enzymatic phenyl containing antioxidant compounds not only serve as phytoanticipins against biotic stresses, also play important role in defense mechanism under abiotic stresses including high temperature stress [83]. Similar findings were reported previously in several other crops such as wheat, maize, sunflower, rice [84–88]. Potassium obtained from KNO_3_ is a compatible osmoprotectant increases the phenyl compounds accumulation in plants raised from KNO_3_ treated seeds which may change the gene expression encodes for stress responsive compounds [89]. Studies also reported significant increase in expression of genes responsible for biosynthesis of stress responsive compounds in maize, wheat, tomato and rice [90–92, 40, 64, 93]. Moreover, physiological performance (photosynthetic rate, stomatal conductance, and water use efficiency) of carrot plants raised from KNO_3_ treated seeds was also modulated positively even under high temperature stress condition in early cropping season. Our findings are in conformity with the findings of other researchers who stated that seed priming improved stomatal conductance and water use efficiency along with transpiration rate and photosynthetic rate under abiotic stress conditions [94–96]. This could be attributed to potential of KNO_3_ treatment resulted in positive up regulation of osmotic balance which enable the regular water uptake and avoid the dehydration. Balanced osmotic and turgor potential facilitate the regulation of stomatal aperture and density and can maintain the CO2 assimilation level without any excessive water loss [97]. Additionally, these osmotic adjustments and positive stomatal regulations enable the plant to continue the sustainable photosynthesis and translocation of photosynthates to the target tissues that is crucial for growth under high temperature stress [98].

Growth of yield contributing root traits was also improved by timely enhanced seedling emergence in response to KNO_3_ seed priming. Previous studies also suggested that potassium nitrate positively regulated the root growth by increasing the root volume and increases the surface area for element absorption ultimately leads to increase in root weight [99]. Additionally, nitrate ions ensure the availability of important nitrogen containing compounds improve the cellular growth and structure. Potassium nitrate treatments also regulate the hormonal production such as auxins, cytokinins, and gibberellins positively while lower the production of abscisic acid in plants under high temperature stress by exogenously providing potassium and nitrate ions required for hormonal synthesis and transport. Phytohormones assist the cell cycle by regular cell divisions, cell elongation, and cell differentiation can induce normal growth and development patterns in stressed plants as well [100]. Use of potassium nitrate in various agricultural products including fertilizers is very common as it improves the soil fertility and crop growth and yield by boosting the accumulation of nutrients like nitrogen, potassium, free amino acids, and proteins [101]. According to previous reports, potassium nitrate improved plant root growth and yield in soybean, cantaloupe [102]. Current study suggests that seed priming with potassium nitrate (50 mM) could lessen the adverse impacts of high temperature stress on carrot plants in early cropping system.

### Mechanistic approach

**Fig. 8:**
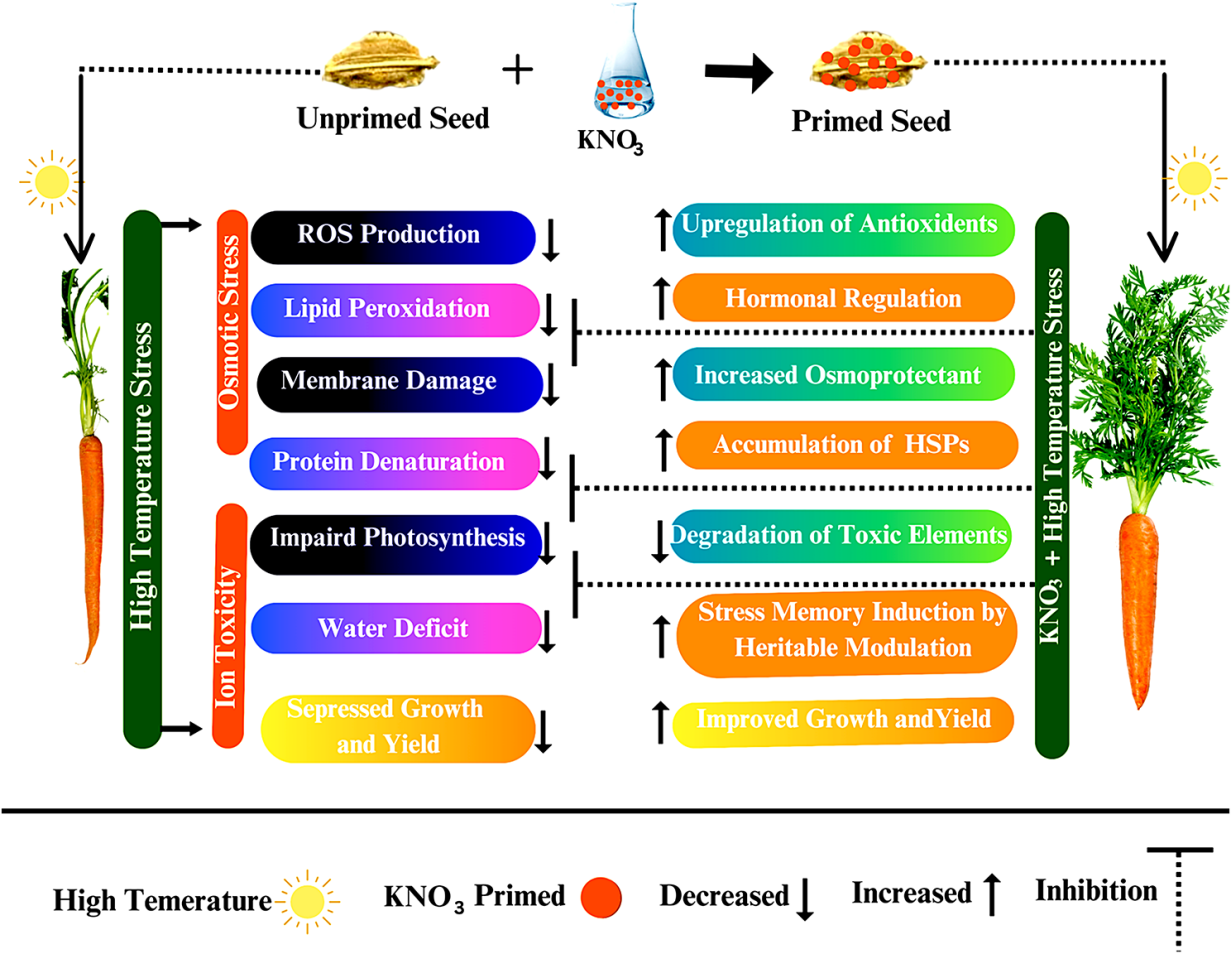
Proposed mechanistic model for the KNO_3_-induced alleviation of adverse effects of high temperature stress in carrot seed

A possible mechanism of KNO_3_ mediated high temperature stress tolerance is presented in Fig. 8 as it involves a complex interplay of several physiological and epigenetic modulations. KNO_3_-mediated seed priming facilitates the seed to imbibe the water supplemented with potassium (K^+^) and nitrate (NO_3_^−^) ions where K^+^ maintains the homeostasis and increases the osmotic potential that prevent the excessive transpiration by making it difficult for water molecules to leave the cells [103]. On the other hand, NO_3_^−^ serve as a nitrogen source for the synthesis of several amino acids such as proline and cysteine as well as stress responsive proteins including heat shock proteins, aminotransferases, and other proteins involve in the degradation of harmful amino acids like lysine [13]. Lysine is an essential amino acid for plant growth and development but high accumulation under stress conditions leads to significant reduction in seed germination, reserve food synthesis and storage, protein denaturation and cell membrane disintegration, and also negative affects the essential amino acid’s balance [104]. High accumulation of lysine resulted in reduced seed germination in Arabidopsis and soyabean [105]. Thus, Osmo-regulation is maybe the first and initial modulation induced by KNO_3_-mediated seed priming in the plant cell under abiotic stress conditions. Moreover, KNO_3_-mediated seed priming triggers the productions of various stress responsive enzymatic and non-enzymatic antioxidants such as catalase, superoxide dismutase, ascorbate peroxidase, phenolics, flavonoids, glutathione reductase by the rapid detoxification of reactive oxygen species and maintain the cell integrity [71, 106]. KNO_3_ also modulates the level and activity of different hormonal responses (indole acetic acid, gibberellins, and ABA) to improve the abiotic stress tolerance in plants [100]. Auxins and cytokinins work antagonistically to regulate the plant responses towards abiotic stress tolerance [107] and exogenous application of KNO_3_ regulate the auxin-cytokinin balance for the better tolerance to abiotic stresses such as drought, salinity and high temperature [108]. Gibberellin is a key signaling molecule interacts with other hormones (ethylene, auxins, salicylic acid, jasmonic acid, and ABA) to regulate the plant growth responses and transcriptional responses such as stomatal regulation, transpiration and photosynthesis rate, and plant defense activities crucial for abiotic tolerance [109]. All these physiological and biochemical modulations are actually induced in plants at molecular level by regulation of several transcriptional factors responsible for gene expression of these metabolites, enzymatic and non-enzymatic antioxidants, and phytohormones [110]. Besides, KNO_3_ also induces stress memory in plants by modulating the level and pattern of DNA methylation, and acetylation and methylation of histones may leads to heritable modification in the chromatin structure and gene expressions crucial to enhance the abiotic stress tolerance [111, 112]. KNO_3_ induced stress memory in plants makes it a promising micro-molecular ameliorant for improving crop resilience towards biotic and abiotic stress conditions.

## Conclusion

In general, seed priming with KNO_3_ at low concentrations enhanced the seed germination and seedling growth while the higher concentrations lowered them under high temperature stress in both in vitro and open field conditions. Seed priming with 50 mM of KNO_3_ was the most effective in reducing the cellular material’s outflow by lowering the EC which could be a possibility of vigor improvement due to less membrane damage. It also resulted in better scavenging of reactive oxygen species by enhanced activities of stress responsive enzymes (peroxidase, catalase, superoxidase dismutase and total antioxidants and phenolics) and improved photosynthetic rate and WUE which led towards better root growth and biomass production under high temperature stress. The current investigation indicates that pre-sowing seed treatments not only enhance seed vigor but also stimulate the growth, yield, and quality of carrot. Besides, KNO_3_ may induce epigenetic and heritable genetic modifications in plants thus, future studies should focus on better understanding of the specific genetic and molecular mechanisms involved in KNO_3_-mediated high temperature stress tolerance in carrots.

### Electronic supplementary material

Below is the link to the electronic supplementary material.

Supplementary Material 1

## Data Availability

The author confirms that all data generated or analyzed during this study are included in this published article. Contact: Dr. Muhammad Mahmood ur Rehman School of Agricultural Engineering, Jiangsu University, China Email: mmrehman@ujs.edu.cn/mahmood2443@gmail.com

## Study protocol must comply with relevant institutional, national, and international guidelines and legislation

Our experiment follows the relevant institutional, national, and international guidelines and legislation.
