# Correction: Seed priming with potassium nitrate alleviates the high temperature stress by modulating growth and antioxidant potential in carrot seeds and seedlings

**DOI:** 10.1186/s12870-024-05414-9

**Published:** 2024-07-18

**Authors:** Muhammad Mahmood ur Rehman, Jizhan Liu, Aneela Nijabat, Ibtisam M. Alsudays, Muneera A. Saleh, Khalid H. Alamer, Houneida Attia, Khurram Ziaf, Qamar uz Zaman, Muhammad Amjad

**Affiliations:** 1 School of Agricultural Engineering, Jiangsu University, Zhenjiang, 212013 China; 2 Institute of Horticultural Sciences, University of Agriculture Faisalabad, Faisalabad, 38040 Pakistan; 3 Department of Botany, University of Mianwali, Mianwali, 42200 Pakistan; 4 Department of Biology, College of Science, Qassim University, Burydah, 52571 Saudi Arabia; 5 Department of Biology, College of Sciences, Taif University, P.O. Box 11099, Taif, 21944 Saudi Arabia; 6 Biological Sciences Department, Faculty of Science and Arts, King Abdulaziz University, Rabigh, 21911 Saudi Arabia; 7 Department of Environmental Sciences, The University of Lahore, Lahore, 54590 Pakistan

**Correction: BMC Plant Biol 24**,** 606 (2024)**

10.1186/s12870-024-05292-1

Following publication of the original article [1], the authors identified an error in the Acknowledgement and Funding sections. The corrections are highlighted in bold. The details are given below:

**Acknowledgements**

Instead of: The authors would like to thank the Jiangsu Funding Program for Excellent Postdoctoral Talent (2024ZB873) for their support and also acknowledge Deanship of Graduate studies and Scientific Research, Taif University, for their contribution in this work.

Correct statement: The authors would like to thank the Jiangsu Funding Program for Excellent Postdoctoral Talent (2024ZB873) for their support and also acknowledge **Deanship of Scientific Research**,** Taif University for funding this work.**

**Funding**

Instead of: The research was supported by the Deanship of Graduate Studies and Scientific Research, Taif University, SA and Jiangsu Funding Program for Excellent Postdoctoral Talent (2024ZB873).

Correct statement: **The researchers would like to acknowledge Deanship of Scientific Research**,** Taif University for funding this work.** SA and Jiangsu Funding Program for Excellent Postdoctoral Talent (2024ZB873).

The original article [1] has been corrected.
