# Supplementary material for: Seed priming with potassium nitrate alleviates the high temperature stress by modulating growth and antioxidant potential in carrot seeds and seedlings
Source: BMC Plant Biol. 2024 Jun 26;24:606. doi: 10.1186/s12870-024-05292-1 (PMC11201870; doi:10.1186/s12870-024-05292-1)
Supplement: Supplementary file 1 — Supplementary Material 1 [file 12870_2024_5292_MOESM1_ESM.docx]

| **Table 1. Seasonal month-wise (whole-sale) prices of carrot in main market-Punjab-Pakistan** | | | | | |
| --- | --- | --- | --- | --- | --- |
| **Market/Year** | **(Early crop)** | **Mid-season crop** | | **(Late crop)** | |
|  | **Nov** | **Dec** | **Jan** | **Feb** | **Mar** |
| **2018-19** | 1194 | 704 | 509 | 514 | 573 |
| **2019-20** | 1274 | 1146 | 1043 | 900 | 973 |
| **2020-21** | 1687 | 1239 | 822 | 713 | 900 |
| **2021-22** | 1525 | 1107 | 1010 | 869 | 1032 |
| **2022-23** | 1992 | 1362 | 1054 | 1216 | 1830 |

(Numerals in each (month) column show the prices of carrot (Rs./ 40 kg), while month of November representing highest carrot root prices that was targeted in present study as early crop (GOP, 2024).

| **Table. 2 Influence of KNO_3_ priming on electrical conductivity EC (µS/cm) of carrot seeds** | | | | | |
| --- | --- | --- | --- | --- | --- |
| **Seed priming treatments** | **Seed soaking durations (hours)** | | | | |
|  | **1 h** | **3 h** | **6 h** | **12 h** | **24 h** |
| **T0** | 764 | 804 | 857 | 1005 | 1088 |
| **T1** | 168 | 195 | 248 | 287 | 340 |
| **T2** | 253 | 295 | 316 | 352 | 369 |
| **T3** | 469 | 513 | 537 | 579 | 565 |
| **T4** | 272 | 314 | 343 | 396 | 443 |
| **T5** | 505 | 556 | 586 | 637 | 677 |
| **T6** | 589 | 634 | 698 | 736 | 797 |
| **T7** | 601 | 714 | 799 | 855 | 912 |
